# Supplementary material for: Income differences in COVID-19 incidence and severity in Finland among people with foreign and native background: A population-based cohort study of individuals nested within households
Source: PLoS Med. 2022 Aug 10;19(8):e1004038. doi: 10.1371/journal.pmed.1004038 (PMC9365184; doi:10.1371/journal.pmed.1004038)
Supplement: S1 Table — (DOCX) [file pmed.1004038.s002.docx]

**S1 Table. The distributions of the population at risk and cases in the incidence and severity analyses by risk factors.**

|  | Incidence analysis | | | | Severity analysis | | | |
| --- | --- | --- | --- | --- | --- | --- | --- | --- |
|  | *Population at risk* | | *Cases* | | *Population at risk* | | *Cases* | |
|  | Full <65 household population | | Clinically confirmed COVID-19 from 1 Jul 2020 to 22 Feb 2021 | | Clinically confirmed COVID-19 from 1 Jul to 31 Dec 2020 | | Severe illness due to COVID-19 from 1 Jul to 31 Dec 2020 | |
| Household income | N | % | N | % | N | % | N | % |
| Quintile 5 | 863 067 | 20.0 | 7 278 | 18.9 | 4 726 | 19.6 | 135 | 21.6 |
| Quintile 4 | 863 068 | 20.0 | 6 922 | 18.0 | 4 227 | 17.5 | 112 | 17.9 |
| Quintile 3 | 863 070 | 20.0 | 6 383 | 16.6 | 3 949 | 16.4 | 107 | 17.1 |
| Quintile 2 | 863 068 | 20.0 | 6 976 | 18.1 | 4 243 | 17.6 | 96 | 15.4 |
| Quintile 1 | 863 069 | 20.0 | 10 908 | 28.4 | 6 993 | 29.0 | 175 | 28.0 |
| Hospital district |  |  |  |  |  |  |  |  |
| Helsinki Metropolitan (HUS) | 1 369 041 | 31.7 | 21 447 | 55.8 | 13 240 | 54.9 | 295 | 47.2 |
| Other | 2 946 301 | 68.3 | 17 020 | 44.2 | 10 898 | 45.1 | 330 | 52.8 |
| Urbanicity |  |  |  |  |  |  |  |  |
| Urban | 2 709 211 | 62.8 | 31 351 | 81.5 | 19 649 | 81.4 | 478 | 76.5 |
| Peri-urban | 717 412 | 16.6 | 3 731 | 9.7 | 2 329 | 9.7 | 58 | 9.3 |
| Rural | 888 719 | 20.6 | 3 385 | 8.8 | 2 160 | 9.0 | 89 | 14.2 |
| Household size |  |  |  |  |  |  |  |  |
| 1 | 731 103 | 16.9 | 5 985 | 15.6 | 3 792 | 15.7 | 119 | 19.0 |
| 2 | 1 172 040 | 27.2 | 9 040 | 23.5 | 5 734 | 23.8 | 223 | 35.7 |
| 3 | 825 635 | 19.1 | 7 199 | 18.7 | 4 480 | 18.6 | 107 | 17.1 |
| 4 | 909 832 | 21.1 | 8 288 | 21.6 | 5 143 | 21.3 | 82 | 13.1 |
| 5+ | 676 732 | 15.7 | 7 955 | 20.7 | 4 989 | 20.7 | 94 | 15.0 |
| Foreign background |  |  |  |  |  |  |  |  |
| Yes | 347 375 | 8.1 | 10 390 | 27.0 | 6 652 | 27.6 | 195 | 31.2 |
| No | 3 967 967 | 91.9 | 28 077 | 73.0 | 17 486 | 72.4 | 430 | 68.8 |
| Household-level work and school exposures | | | | | | | | |
| Lower non-manual employee | 1 833 811 | 42.5 | 15 703 | 40.8 |  |  |  |  |
| Self-employed | 571 184 | 13.2 | 4 475 | 11.6 |  |  |  |  |
| Manual worker | 1 436 095 | 33.3 | 12 909 | 33.6 |  |  |  |  |
| Student in secondary or tertiary level | 1 477 725 | 34.2 | 16 773 | 43.6 |  |  |  |  |
| Child aged 13–15 | 681 113 | 15.8 | 7 440 | 19.3 |  |  |  |  |
| Child aged 7–12 | 1 125 257 | 26.1 | 10 822 | 28.1 |  |  |  |  |
| Child aged <7 | 961 252 | 22.3 | 8 860 | 23.0 |  |  |  |  |
| Comorbidities |  |  |  |  |  |  |  |  |
| Cancer |  |  |  |  | 162 | 0.7 | 18 | 2.9 |
| Kidney failure |  |  |  |  | 20 | 0.1 | 8 | 1.3 |
| Chronic lung disease |  |  |  |  | 789 | 3.3 | 64 | 10.2 |
| Diabetes |  |  |  |  | 787 | 3.3 | 92 | 14.7 |
| Chronic heart disease |  |  |  |  | 512 | 2.1 | 71 | 11.4 |
| Psychotic disorders |  |  |  |  | 173 | 0.7 | 12 | 1.9 |
| Personal occupation |  |  |  |  |  |  |  |  |
| Upper non-manual employee |  |  |  |  | 3 809 | 15.8 | 79 | 12.6 |
| Lower non-manual employee |  |  |  |  | 5 773 | 23.9 | 121 | 19.4 |
| Self-employed |  |  |  |  | 1 483 | 6.1 | 44 | 7.0 |
| Manual worker |  |  |  |  | 4 671 | 19.4 | 111 | 17.8 |
| Student |  |  |  |  | 3 500 | 14.5 | 26 | 4.2 |
| Pensioner |  |  |  |  | 687 | 2.9 | 115 | 18.4 |
| Other/Unknown |  |  |  |  | 4 215 | 17.5 | 129 | 20.6 |
| Total N | 4 315 342 | 100 | 38 467 | 100 | 24 138 | 100 | 625 | 100 |
