# Supplementary material for: Income differences in COVID-19 incidence and severity in Finland among people with foreign and native background: A population-based cohort study of individuals nested within households
Source: PLoS Med. 2022 Aug 10;19(8):e1004038. doi: 10.1371/journal.pmed.1004038 (PMC9365184; doi:10.1371/journal.pmed.1004038)
Supplement: S2 Table — Hospitalization is defined as any admission to the hospital with a COVID-19 diagnosis. Results are from 2-level logistic regressions, with individuals at level 1 nested in households at level 2. All models are adjusted for age and age squared and sex. (DOCX) [file pmed.1004038.s003.docx]

**S2 Table. Odds ratios of hospitalization with COVID-19 diagnosis (N=696) among those infected from 1 July to 31 December 2020 (N=24 138), individuals living in under-65 households.**

|  | Models | | | | | |
| --- | --- | --- | --- | --- | --- | --- |
|  | 1* | 2 | 3 | 4 | 5 | 6 |
| 1. Household income (ref. 5)  *(reference* | OR (95% CI) | OR (95% CI) | OR (95% CI) | OR (95% CI) | OR (95% CI) | OR (95% CI) |
| Quintile 4 | 1.03 (0.79–1.33) | 1.01 (0.77–1.31) | 1.01 (0.77–1.32) | 0.96 (0.73–1.26) | 0.98 (0.75–1.27) | 0.95 (0.72–1.24) |
|  | p=0.85 | p=0.97 | p=0.94 | p=0.78 | p=0.86 | p=0.69 |
| Quintile 3 | 1.21 (0.92–1.57) | 1.16 (0.88–1.52) | 1.16 (0.88–1.52) | 1.07 (0.81–1.42) | 1.10 (0.84–1.45) | 1.03 (0.78–1.37) |
|  | p=0.17 | p=0.29 | p=0.29 | p=0.63 | p=0.49 | p=0.82 |
| Quintile 2 | 1.22 (0.93–1.61) | 1.14 (0.86–1.50) | 1.13 (0.85–1.50) | 1.00 (0.75–1.34) | 1.03 (0.78–1.37) | 0.92 (0.68–1.25) |
|  | p=0.15 | p=0.37 | p=0.40 | p=1.00 | p=0.82 | p=0.61 |
| Quintile 1 (lowest) | 1.88 (1.47–2.40) | 1.78 (1.38–2.28) | 1.74 (1.34–2.25) | 1.41 (1.06–1.88) | 1.45 (1.10–1.91) | 1.15 (0.84–1.58) |
|  | p<0.001 | p<0.001 | p<0.001 | p=0.02 | p=0.008 | p=0.38 |
| 2. Hospital district (ref. other) |  |  |  |  |  |  |
| Helsinki Metropolitan (HUS) | 0.78 (0.65–0.93) | 0.79 (0.66–0.94) | 0.79 (0.66–0.95) | 0.79 (0.66–0.95) | 0.76 (0.63–0.91) | 0.76 (0.63–0.92) |
|  | p=0.005 | p=0.009 | p=0.01 | p=0.01 | p=0.003 | p=0.004 |
| 3. Urbanicity (ref. rural) |  |  |  |  |  |  |
| Urban | 0.96 (0.74–1.25) | 0.89 (0.68–1.17) | 0.89 (0.68–1.17) | 0.89 (0.68–1.18) | 0.85 (0.65–1.12) | 0.84 (0.64–1.11) |
|  | p=0.78 | p=0.41 | p=0.41 | p=0.43 | p=0.25 | p=0.23 |
| Peri-urban | 0.71 (0.50–1.01) | 0.71 (0.50–1.02) | 0.71 (0.50–1.02) | 0.71 (0.50–1.02) | 0.71 (0.50–1.02) | 0.72 (0.50–1.03) |
|  | p=0.05 | p=0.06 | p=0.06 | p=0.07 | p=0.06 | p=0.07 |
| 4. Comorbidities |  |  |  |  |  |  |
| a) Cancer | 1.81 (1.06–3.11) | 1.65 (0.94–2.90) | 1.65 (0.94–2.90) | 1.65 (0.93–2.93) | 1.68 (0.95–2.95) | 1.67 (0.94–2.95) |
|  | p=0.03 | p=0.08 | p=0.08 | p=0.09 | p=0.07 | p=0.08 |
| b) Kidney failure | 16.92 (5.41–52.91) | 9.31 (2.80–30.91) | 9.10 (2.74–30.22) | 8.05 (2.39–27.05) | 9.26 (2.79–30.74) | 7.78 (2.32–26.09) |
|  | p<0.001 | p<0.001 | p<0.001 | p=0.001 | p<0.001 | p=0.001 |
| c) Chronic lung disease | 2.37 (1.77–3.18) | 2.35 (1.74–3.17) | 2.34 (1.73–3.17) | 2.31 (1.70–3.13) | 2.44 (1.80–3.31) | 2.39 (1.76–3.25) |
|  | p<0.001 | p<0.001 | p<0.001 | p<0.001 | p<0.001 | p<0.001 |
| d) Diabetes | 2.19 (1.67–2.87) | 1.79 (1.35–2.38) | 1.80 (1.35–2.38) | 1.76 (1.32–2.34) | 1.76 (1.33–2.34) | 1.74 (1.31–2.31) |
|  | p<0.001 | p<0.001 | p<0.001 | p<0.001 | p<0.001 | p<0.001 |
| e) Chronic heart disease | 1.82 (1.35–2.46) | 1.37 (0.99–1.89) | 1.37 (0.99–1.89) | 1.34 (0.96–1.86) | 1.42 (1.03–1.96) | 1.38 (1.00–1.92) |
|  | p<0.001 | p=0.06 | p=0.06 | p=0.08 | p=0.04 | p=0.05 |
| f) Psychotic disorders | 1.81 (0.94–3.49) | 1.38 (0.71–2.72) | 1.37 (0.70–2.71) | 1.01 (0.50–2.02) | 1.52 (0.77–2.99) | 1.07 (0.53–2.15) |
|  | p=0.07 | p=0.34 | p=0.36 | p=0.99 | p=0.23 | p=0.85 |
| 5. Household size (ref. 1) |  |  |  |  |  |  |
| 2 | 0.89 (0.70–1.13) |  | 0.98 (0.77–1.26) |  |  | 0.90 (0.70–1.16) |
|  | p=0.33 |  | p=0.89 |  |  | p=0.43 |
| 3 | 0.80 (0.61–1.05) |  | 0.89 (0.67–1.17) |  |  | 0.81 (0.61–1.07) |
|  | p=0.11 |  | p=0.39 |  |  | p=0.14 |
| 4 | 0.77 (0.58–1.03) |  | 0.86 (0.64–1.15) |  |  | 0.78 (0.58–1.06) |
|  | p=0.08 |  | p=0.30 |  |  | p=0.11 |
| 5+ | 1.06 (0.80–1.40) |  | 1.03 (0.77–1.37) |  |  | 0.90 (0.67–1.21) |
|  | p=0.70 |  | p=0.84 |  |  | p=0.48 |
| 6. Occupation (ref. upper non-manual) | | | | | | |
| Lower non-manual | 1.00 (0.75–1.32) |  |  | 0.94 (0.70–1.25) |  | 0.92 (0.69–1.24) |
|  | p=0.99 |  |  | p=0.66 |  | p=0.59 |
| Self-employed | 1.08 (0.74–1.58) |  |  | 1.01 (0.68–1.48) |  | 0.98 (0.66–1.45) |
|  | p=0.69 |  |  | p=0.98 |  | p=0.92 |
| Manual worker | 1.14 (0.85–1.52) |  |  | 1.07 (0.79–1.45) |  | 0.98 (0.72–1.34) |
|  | p=0.38 |  |  | p=0.67 |  | p=0.92 |
| Student | 1.16 (0.76–1.78) |  |  | 0.97 (0.62–1.52) |  | 0.90 (0.57–1.41) |
|  | p=0.49 |  |  | p=0.89 |  | p=0.65 |
| Pensioner | 3.15 (2.18–4.55) |  |  | 2.38 (1.60–3.56) |  | 2.34 (1.56–3.50) |
|  | p<0.001 |  |  | p<0.001 |  | p<0.001 |
| Other/Unknown | 1.73 (1.30–2.29) |  |  | 1.35 (0.98–1.88) |  | 1.22 (0.88–1.71) |
|  | p<0.001 |  |  | p=0.07 |  | p=0.23 |
| 7. Foreign background (ref. no) |  |  |  |  |  |  |
| Yes | 1.64 (1.36–1.98) |  |  |  | 1.46 (1.17–1.82) | 1.51 (1.20–1.90) |
|  | p<0.001 |  |  |  | p=0.001 | p<0.001 |
| Household ICC | 0.167 | 0.185 | 0.185 | 0.197 | 0.184 | 0.192 |
|  | (0.064–0.371) | (0.078–0.380) | (0.078–0.380) | (0.088–0.382) | (0.077–0.381) | (0.084–0.380) |
| Ref. = Reference category, OR = Odds ratio, CI = Confidence interval, p = p-value, ICC = Intra-class correlation | | | | | | |
| * Each variable adjusted separately for age and age squared, sex, hospital district and urbanicity | | | | | | |
| † Calculated from a model including age and age squared, sex, hospital district and urbanicity | | | | | | |
