# Supplementary material for: Income differences in COVID-19 incidence and severity in Finland among people with foreign and native background: A population-based cohort study of individuals nested within households
Source: PLoS Med. 2022 Aug 10;19(8):e1004038. doi: 10.1371/journal.pmed.1004038 (PMC9365184; doi:10.1371/journal.pmed.1004038)
Supplement: S3 Table — Severe illness is defined as having at least 3 consecutive days of inpatient care with a COVID-19 diagnosis. Results are from 2-level logistic regressions, with individuals at level 1 nested in households at level 2. All models are adjusted for age and age squared and sex. (DOCX) [file pmed.1004038.s004.docx]

**S3 Table. Odds ratios of severe illness with COVID-19 as the primary diagnosis (N=387) among those infected from 1 July to 31 December 2020 (N=24 138), individuals living in under-65 households.**

|  | Models | | | | | |
| --- | --- | --- | --- | --- | --- | --- |
|  | 1* | 2 | 3 | 4 | 5 | 6 |
| 1. Household income (ref. 5)  *(reference* | OR (95% CI) | OR (95% CI) | OR (95% CI) | OR (95% CI) | OR (95% CI) | OR (95% CI) |
| Quintile 4 | 0.92 (0.65–1.31) | 0.90 (0.64–1.28) | 0.91 (0.64–1.29) | 0.87 (0.61–1.25) | 0.87 (0.61–1.24) | 0.85 (0.59–1.23) |
|  | p=0.65 | p=0.57 | p=0.58 | p=0.46 | p=0.45 | p=0.40 |
| Quintile 3 | 1.36 (0.97–1.91) | 1.31 (0.93–1.84) | 1.30 (0.92–1.83) | 1.22 (0.85–1.75) | 1.24 (0.88–1.74) | 1.16 (0.81–1.67) |
|  | p=0.08 | p=0.12 | p=0.14 | p=0.27 | p=0.23 | p=0.42 |
| Quintile 2 | 1.20 (0.84–1.72) | 1.11 (0.77–1.60) | 1.08 (0.74–1.56) | 0.97 (0.66–1.43) | 1.00 (0.69–1.45) | 0.87 (0.58–1.29) |
|  | p=0.31 | p=0.56 | p=0.70 | p=0.90 | p=1.00 | p=0.48 |
| Quintile 1 (lowest) | 1.89 (1.37–2.59) | 1.79 (1.30–2.47) | 1.67 (1.20–2.33) | 1.36 (0.93–1.97) | 1.43 (1.00–2.04) | 1.03 (0.68–1.56) |
|  | p<0.001 | p<0.001 | p=0.003 | p=0.11 | p=0.05 | p=0.88 |
| 2. Hospital district (ref. other) |  |  |  |  |  |  |
| Helsinki Metropolitan (HUS) | 0.86 (0.68–1.08) | 0.87 (0.69–1.10) | 0.87 (0.69–1.10) | 0.88 (0.69–1.11) | 0.84 (0.66–1.06) | 0.84 (0.66–1.07) |
|  | p=0.20 | p=0.26 | p=0.26 | p=0.28 | p=0.14 | p=0.16 |
| 3. Urbanicity (ref. rural) |  |  |  |  |  |  |
| Urban | 0.95 (0.68–1.34) | 0.89 (0.63–1.26) | 0.89 (0.63–1.26) | 0.89 (0.62–1.27) | 0.84 (0.59–1.20) | 0.83 (0.58–1.20) |
|  | p=0.79 | p=0.51 | p=0.51 | p=0.52 | p=0.34 | p=0.33 |
| Peri-urban | 0.75 (0.48–1.18) | 0.76 (0.48–1.20) | 0.76 (0.48–1.20) | 0.76 (0.48–1.21) | 0.76 (0.48–1.21) | 0.76 (0.48–1.22) |
|  | p=0.21 | p=0.24 | p=0.23 | p=0.25 | p=0.24 | p=0.26 |
| 4. Comorbidities |  |  |  |  |  |  |
| a) Cancer | 2.15 (1.13–4.08) | 1.96 (1.02–3.77) | 1.97 (1.02–3.81) | 1.98 (1.01–3.86) | 2.00 (1.03–3.86) | 2.00 (1.02–3.93) |
|  | p=0.02 | p=0.04 | p=0.04 | p=0.05 | p=0.04 | p=0.04 |
| b) Kidney failure | 5.93 (1.38–25.45) | 2.88 (0.64–13.08) | 2.75 (0.60–12.57) | 2.48 (0.53–11.62) | 2.87 (0.63–13.19) | 2.32 (0.49–11.04) |
|  | p=0.02 | p=0.17 | p=0.19 | p=0.25 | p=0.18 | p=0.29 |
| c) Chronic lung disease | 2.37 (1.64–3.44) | 2.30 (1.58–3.36) | 2.31 (1.58–3.37) | 2.27 (1.54–3.33) | 2.41 (1.65–3.52) | 2.36 (1.60–3.49) |
|  | p<0.001 | p<0.001 | p<0.001 | p<0.001 | p<0.001 | p<0.001 |
| d) Diabetes | 1.97 (1.40–2.76) | 1.61 (1.13–2.29) | 1.61 (1.13–2.30) | 1.58 (1.10–2.26) | 1.58 (1.11–2.25) | 1.56 (1.09–2.24) |
|  | p<0.001 | p=0.008 | p=0.008 | p=0.01 | p=0.01 | p=0.02 |
| e) Chronic heart disease | 1.75 (1.20–2.55) | 1.38 (0.93–2.06) | 1.39 (0.93–2.07) | 1.35 (0.90–2.03) | 1.44 (0.97–2.15) | 1.40 (0.93–2.11) |
|  | p=0.003 | p=0.11 | p=0.11 | p=0.15 | p=0.07 | p=0.11 |
| f) Psychotic disorders | 2.17 (1.00–4.75) | 1.69 (0.76–3.73) | 1.73 (0.78–3.85) | 1.19 (0.52–2.72) | 1.88 (0.84–4.17) | 1.31 (0.57–3.01) |
|  | p=0.05 | p=0.20 | p=0.18 | p=0.67 | p=0.12 | p=0.53 |
| 5. Household size (ref. 1) |  |  |  |  |  |  |
| 2 | 0.88 (0.64–1.20) |  | 0.98 (0.71–1.35) |  |  | 0.89 (0.64–1.24) |
|  | p=0.42 |  | p=0.91 |  |  | p=0.48 |
| 3 | 0.72 (0.50–1.04) |  | 0.81 (0.56–1.17) |  |  | 0.73 (0.50–1.07) |
|  | p=0.08 |  | p=0.26 |  |  | p=0.10 |
| 4 | 0.71 (0.49–1.05) |  | 0.79 (0.53–1.17) |  |  | 0.71 (0.48–1.06) |
|  | p=0.09 |  | p=0.24 |  |  | p=0.09 |
| 5+ | 1.29 (0.90–1.84) |  | 1.28 (0.89–1.83) |  |  | 1.10 (0.76–1.61) |
|  | p=0.16 |  | p=0.18 |  |  | p=0.61 |
| 6. Occupation (ref. upper non-manual) | | | | | | |
| Lower non-manual | 0.88 (0.60–1.28) |  |  | 0.82 (0.56–1.21) |  | 0.82 (0.55–1.20) |
|  | p=0.49 |  |  | p=0.32 |  | p=0.31 |
| Self-employed | 1.05 (0.64–1.71) |  |  | 0.97 (0.59–1.61) |  | 0.95 (0.57–1.57) |
|  | p=0.85 |  |  | p=0.92 |  | p=0.84 |
| Manual worker | 1.05 (0.72–1.53) |  |  | 0.98 (0.66–1.46) |  | 0.90 (0.60–1.36) |
|  | p=0.80 |  |  | p=0.92 |  | p=0.62 |
| Student | 1.20 (0.68–2.14) |  |  | 1.02 (0.55–1.86) |  | 0.94 (0.51–1.73) |
|  | p=0.53 |  |  | p=0.96 |  | p=0.84 |
| Pensioner | 3.25 (2.04–5.18) |  |  | 2.46 (1.48–4.09) |  | 2.44 (1.46–4.09) |
|  | p<0.001 |  |  | p=0.001 |  | p=0.001 |
| Other/Unknown | 1.73 (1.19–2.49) |  |  | 1.39 (0.91–2.11) |  | 1.26 (0.82–1.94) |
|  | p=0.004 |  |  | p=0.13 |  | p=0.29 |
| 7. Foreign background (ref. no) |  |  |  |  |  |  |
| Yes | 1.70 (1.33–2.18) |  |  |  | 1.53 (1.15–2.03) | 1.54 (1.14–2.09) |
|  | p<0.001 |  |  |  | p=0.003 | p=0.005 |
| Household ICC | 0.200† | 0.195 | 0.202 | 0.224 | 0.202 | 0.232 |
|  | (0.061–0.492) | (0.057–0.494) | (0.063–0.488) | (0.080–0.491) | (0.062–0.492) | (0.087–0.488) |
| Ref. = Reference category, OR = Odds ratio, CI = Confidence interval, p = p-value, ICC = Intra-class correlation | | | | | | |
| * Each variable adjusted separately for age and age squared, sex, hospital district and urbanicity | | | | | | |
| † Calculated from a model including age and age squared, sex, hospital district and urbanicity | | | | | | |
