# Supplementary material for: Income differences in COVID-19 incidence and severity in Finland among people with foreign and native background: A population-based cohort study of individuals nested within households
Source: PLoS Med. 2022 Aug 10;19(8):e1004038. doi: 10.1371/journal.pmed.1004038 (PMC9365184; doi:10.1371/journal.pmed.1004038)
Supplement: S4 Table — Severe illness is defined as having at least 3 consecutive days of inpatient care with a COVID-19 diagnosis. Results are from 1-level logistic regressions with household-clustered standard errors. All models are adjusted for age and age squared and sex. (DOCX) [file pmed.1004038.s005.docx]

**S4 Table. Odds ratios of severe COVID-19 illness (N=636) from 1 July to 31 December 2020, among all individuals living in under-65 households (N=4 315 342).**

|  | Models | | | | | |
| --- | --- | --- | --- | --- | --- | --- |
|  | 1* | 2 | 3 | 4 | 5 | 6 |
| 1. Household income (ref. 5)  *(reference* | OR (95% CI) | OR (95% CI) | OR (95% CI) | OR (95% CI) | OR (95% CI) | OR (95% CI) |
| Quintile 4 | 1.12 (0.86–1.45) | 1.10 (0.85–1.42) | 1.09 (0.84–1.42) | 1.03 (0.79–1.35) | 0.99 (0.76–1.28) | 0.95 (0.73–1.24) |
|  | p=0.39 | p=0.49 | p=0.52 | p=0.82 | p=0.94 | p=0.70 |
| Quintile 3 | 1.28 (0.99–1.66) | 1.24 (0.96–1.61) | 1.22 (0.94–1.58) | 1.13 (0.86–1.48) | 1.04 (0.80–1.35) | 0.96 (0.73–1.26) |
|  | p=0.06 | p=0.10 | p=0.14 | p=0.37 | p=0.79 | p=0.79 |
| Quintile 2 | 1.34 (1.01–1.76) | 1.28 (0.97–1.69) | 1.23 (0.93–1.63) | 1.12 (0.84–1.50) | 0.96 (0.73–1.26) | 0.86 (0.64–1.15) |
|  | p=0.04 | p=0.08 | p=0.15 | p=0.44 | p=0.76 | p=0.30 |
| Quintile 1 (lowest) | 2.50 (1.98–3.16) | 2.42 (1.91–3.06) | 2.58 (2.00–3.31) | 1.92 (1.45–2.54) | 1.47 (1.17–1.86) | 1.27 (0.96–1.69) |
|  | p<0.001 | p<0.001 | p<0.001 | p<0.001 | p=0.001 | p=0.10 |
| 2. Hospital district (ref. other) |  |  |  |  |  |  |
| Helsinki Metropolitan (HUS) | 1.75 (1.47–2.07) | 1.85 (1.56–2.19) | 1.80 (1.52–2.14) | 1.85 (1.56–2.20) | 1.45 (1.21–1.74) | 1.44 (1.19–1.73) |
|  | p<0.001 | p<0.001 | p<0.001 | p<0.001 | p<0.001 | p<0.001 |
| 3. Urbanicity (ref. rural) |  |  |  |  |  |  |
| Urban | 1.73 (1.36–2.22) | 1.76 (1.37–2.25) | 1.88 (1.46–2.41) | 1.76 (1.37–2.25) | 1.51 (1.18–1.94) | 1.56 (1.21–2.01) |
|  | p<0.001 | p<0.001 | p<0.001 | p<0.001 | p=0.001 | p=0.001 |
| Peri-urban | 0.81 (0.58–1.14) | 0.85 (0.61–1.20) | 0.87 (0.62–1.21) | 0.85 (0.61–1.20) | 0.85 (0.61–1.19) | 0.85 (0.61–1.20) |
|  | p=0.23 | p=0.36 | p=0.40 | p=0.36 | p=0.34 | p=0.36 |
| 4. Comorbidities |  |  |  |  |  |  |
| a) Cancer | 1.58 (1.00–2.50) | 1.55 (0.99–2.45) | 1.57 (1.00–2.48) | 1.56 (0.99–2.45) | 1.63 (1.04–2.58) | 1.63 (1.03–2.58) |
|  | p=0.05 | p=0.06 | p=0.05 | p=0.06 | p=0.04 | p=0.04 |
| b) Kidney failure | 6.94 (3.43–14.03) | 4.29 (2.36–10.26) | 4.95 (2.37–10.32) | 4.93 (2.35–10.31) | 4.61 (2.21–9.64) | 4.46 (2.13–9.34) |
|  | p<0.001 | p<0.001 | p<0.001 | p<0.001 | p<0.001 | p<0.001 |
| c) Chronic lung disease | 2.05 (1.58–2.67) | 1.97 (1.52–2.56) | 2.00 (1.54–2.60) | 1.97 (1.52–2.56) | 2.34 (1.80–3.05) | 2.33 (1.78–3.03) |
|  | p<0.001 | p<0.001 | p<0.001 | p<0.001 | p<0.001 | p<0.001 |
| d) Diabetes | 2.23 (1.77–2.81) | 1.99 (1.54–2.55) | 2.02 (1.57–2.61) | 1.96 (1.53–2.52) | 2.01 (1.56–2.58) | 1.98 (1.54–2.56) |
|  | p<0.001 | p<0.001 | p<0.001 | p<0.001 | p<0.001 | p<0.001 |
| e) Chronic heart disease | 1.31 (1.01–1.71) | 0.95 (0.71–1.28) | 0.97 (0.72–1.30) | 0.95 (0.71–1.27) | 1.06 (0.79–1.43) | 1.06 (0.79–1.42) |
|  | p=0.04 | p=0.76 | p=0.85 | p=0.74 | p=0.69 | p=0.71 |
| f) Psychotic disorders | 0.93 (0.52–1.65) | 0.59 (0.33–1.06) | 0.67 (0.37–1.21) | 0.60 (0.33–1.08) | 0.80 (0.45–1.44) | 0.80 (0.44–1.46) |
|  | p=0.80 | p=0.08 | p=0.19 | p=0.09 | p=0.46 | p=0.47 |
| 5. Household size (ref. 1) |  |  |  |  |  |  |
| 2 | 1.04 (0.83–1.30) |  | 1.37 (1.08–1.75) |  |  | 1.12 (0.88–1.42) |
|  | p=0.74 |  | p=0.01 |  |  | p=0.37 |
| 3 | 1.06 (0.81–1.39) |  | 1.41 (1.06–1.87) |  |  | 1.09 (0.82–1.44) |
|  | p=0.66 |  | p=0.02 |  |  | p=0.56 |
| 4 | 1.10 (0.82–1.46) |  | 1.49 (1.10–2.01) |  |  | 1.16 (0.86–1.56) |
|  | p=0.53 |  | p=0.01 |  |  | p=0.34 |
| 5+ | 2.30 (1.74–3.06) |  | 2.77 (2.06–3.71) |  |  | 1.91 (1.43–2.55) |
|  | p<0.001 |  | p<0.001 |  |  | p<0.001 |
| 6. Occupation (ref. upper non-manual) | | | | | | |
| Lower non-manual | 1.24 (0.93–1.64) |  |  | 1.16 (0.87–1.54) |  | 1.23 (0.92–1.63) |
|  | p=0.14 |  |  | p=0.33 |  | p=0.17 |
| Self-employed | 1.36 (0.94–1.98) |  |  | 1.20 (0.82–1.76) |  | 1.12 (0.76–1.64) |
|  | p=0.10 |  |  | p=0.35 |  | p=0.57 |
| Manual worker | 1.42 (1.06–1.91) |  |  | 1.28 (0.94–1.75) |  | 1.08 (0.79–1.47) |
|  | p=0.02 |  |  | p=0.12 |  | p=0.65 |
| Student | 1.90 (1.19–3.01) |  |  | 1.35 (0.83–2.20) |  | 1.11 (0.69–1.80) |
|  | p=0.007 |  |  | p=0.22 |  | p=0.66 |
| Pensioner | 1.99 (1.43–2.77) |  |  | 1.38 (0.96–1.98) |  | 1.41 (0.98–2.03) |
|  | p<0.001 |  |  | p=0.08 |  | p=0.07 |
| Other/Unknown | 2.44 (1.84–3.24) |  |  | 1.67 (1.21–2.32) |  | 1.40 (1.02–1.93) |
|  | p<0.001 |  |  | p=0.002 |  | p=0.04 |
| 7. Foreign background (ref. no) |  |  |  |  |  |  |
| Yes | 5.49 (4.55–6.61) |  |  |  | 5.21 (4.30–6.30) | 4.88 (4.00–5.95) |
|  | p<0.001 |  |  |  | p<0.001 | p<0.001 |
| Ref. = Reference category, OR = Odds ratio, CI = Confidence interval, p = p-value | | | | | | |
| * Each variable adjusted separately for age and age squared, sex, hospital district and urbanicity | | | | | | |
