# Supplementary figures and images for: Income differences in COVID-19 incidence and severity in Finland among people with foreign and native background: A population-based cohort study of individuals nested within households
Source: PLoS Med. 2022 Aug 10;19(8):e1004038. doi: 10.1371/journal.pmed.1004038 (PMC9365184; doi:10.1371/journal.pmed.1004038)

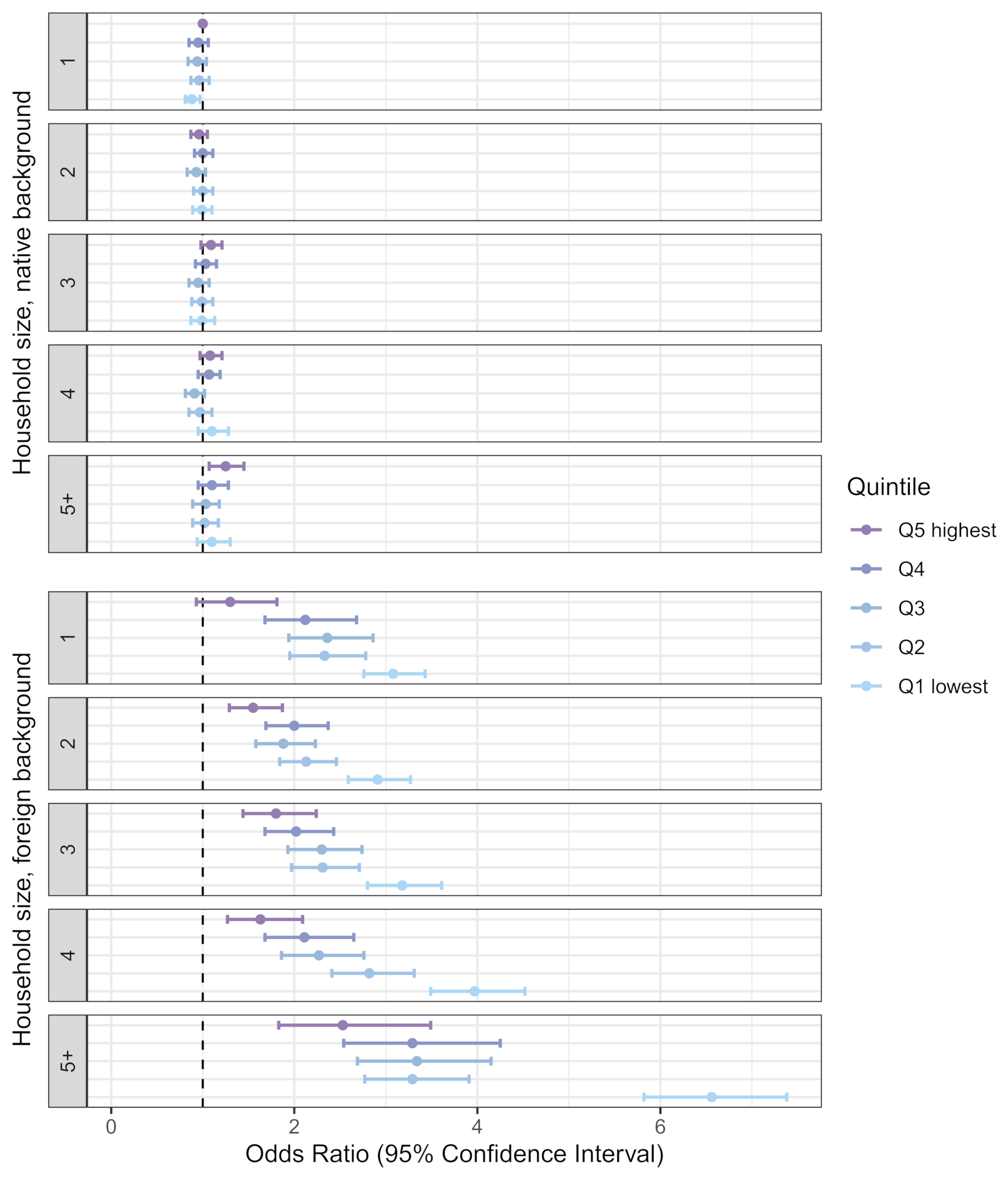

Supplement: S1 Fig — Adjusted for age and age squared, sex, regional characteristics, and household-level work and school exposures. (TIF) [file pmed.1004038.s006.tif]
