# Supplementary material for: Income differences in COVID-19 incidence and severity in Finland among people with foreign and native background: A population-based cohort study of individuals nested within households
Source: PLoS Med. 2022 Aug 10;19(8):e1004038. doi: 10.1371/journal.pmed.1004038 (PMC9365184; doi:10.1371/journal.pmed.1004038)
Supplement: Attachment — Submitted filename: Response_to_reviewers.docx [file pmed.1004038.s007.docx]

**Response to editors and reviewers**

We thank the reviewers for the comments on our manuscript and the thoughtful suggestions for improvement. Please find below our point-by-point response to each of the issues raised by the editors and the reviewers.

Requests from the editors:

1)      Please revise your title according to PLOS Medicine's style. Your title must be nondeclarative and not a question. It should begin with main concept if possible. Please place the study design in the subtitle (i.e., after a colon), e.g., cross-sectional population-based study. Include country in the title. For example, “ Income differences, COVID-19 incidence and severity in Finland: A cross-sectional population-based study”

**Response: We have changed the title into “Income differences in COVID-19 incidence and severity in Finland: a population-based cohort study of individuals nested in households”.**

2)      The Data Availability Statement (DAS) requires revision. For each data source used in your study:
a) If the data are freely or publicly available, state the location of the data: within the paper, in Supporting Information files, or in a public repository (include the DOI or accession number).
b) If the data are owned by a third party but freely available upon request, please note this and state the owner of the data set and contact information for data requests (web or email address). Note that a study author cannot be the contact person for the data.
c) If the data are not freely available, please describe briefly the ethical, legal, or contractual restriction that prevents you from sharing it. Please also include an appropriate contact (web or email address) for inquiries (again, this cannot be a study author).

**Response: We revised our Data Availability Statement to comply with option c) above. It now states the following:** **“Due to data protection regulations of the national register-holders providing the data, we are not allowed to make the data available to third parties. Interested researchers have the possibility to obtain data access by contacting the following register-holding public institutions:**

**• Statistics Finland (http://www.stat.fi/tup/mikroaineistot/index_en.html). Contact by email tutkijapalvelut(at)stat.fi.**

**• Findata (https://findata.fi/en/). Contact by email info(at)findata.fi.“**

3)      Please strongly consider reviewer #3’s comments on your conceptualization of the research question and analyses. The use of DAGs and mediation analyses to untangle causal associations will be critical to refining and strengthening your findings and conclusions. Of note, “foreign background” is not unrelated to ethnicity and this has been explored in other studies.

**Response: We have carefully considered the comments of reviewer #3 regarding our research questions and design and have taken them into account within the scope of our study. We drew DAGs to illustrate the hypothesized causal relationships regarding our key question of interest, i.e. the association between household income and COVID-19 outcomes. As shown in the DAGs (on p. 9 below), we did not have mediators for this association in our analyses, as all our covariates conceptually or temporally preceded household income. Thus a formal mediation analysis, as suggested by Reviewer #3, was not suitable to the research aims we had. We thus retain our current analytical strategy, but have framed it more strongly in modern causal epidemiological terms. We have now clarified the role of all our covariates as confounders in the section on analytical strategy and across the manuscript. We also more thoroughly assessed the interplay between income and foreign background as suggested by Reviewer #3, which revealed a strong income gradient in COVID-19 incidence among people with a foreign background but no association at all between income and COVID-19 incidence in the native population. This is a novel contribution to the literature and suggests that low household income may not be an independent risk factor for COVID-19 infection but, in fact, reflects the differential clustering of other risk factors, foreign background in particular, across income groups. Please also see our more detailed response to the comments of reviewer #3.**

**We agree that ethnicity and foreign background are related concepts. We thus address the previous studies on ethnicity in the Introduction and Discussion, and discuss how their results relate to ours.**

4)      Abstract:
a)      Please combine the Methods and Findings sections into one section, “Methods and Findings”
b)      Please ensure that all numbers presented in the abstract are present and identical to numbers presented in the main manuscript text.
c)      Please include the actual amounts and/or absolute risk(s) of relevant outcomes, not just coefficients.
d)      Please quantify the main results (please present both 95% CIs and p values).
e)      In the last sentence of the Abstract Methods and Findings section, please describe the main limitation(s) of the study's methodology.

**Response: We have revised the Abstract according to the points a)-e) and hope it now reads better.**

5)      At this stage, we ask that you include a short, non-technical Author Summary of your research to make findings accessible to a wide audience that includes both scientists and non-scientists. The Author Summary should immediately follow the Abstract in your revised manuscript. This text is subject to editorial change and should be distinct from the scientific abstract. Please see our author guidelines for more information: <https://journals.plos.org/plosmedicine/s/revising-your-manuscript#loc-author-summary>

**Response: We have included an Author Summary in the revised manuscript.**

6)      Please avoid assertions of primacy (“This is the first study to ..."). Instead, state “to our knowledge” or similar.

**Response: We have revised our wording to avoid assertions of primacy.**

7)      Please remove the last sentence of your introduction (“We adjust our analyses …”) and move it the statistical analysis section

**Response: We have removed the last sentence of the introduction.**

8)      Did your study have a prospective protocol or analysis plan? Please state this (either way) early in the Methods section.
a)      If a prospective analysis plan (from your funding proposal, IRB or other ethics committee submission, study protocol, or other planning document written before analyzing the data) was used in designing the study, please include the relevant prospectively written document with your revised manuscript as a Supporting Information file to be published alongside your study, and cite it in the Methods section. A legend for this file should be included at the end of your manuscript.
b)      If no such document exists, please make sure that the Methods section transparently describes when analyses were planned, and when/why any data-driven changes to analyses took place.
c)      In either case, changes in the analysis-- including those made in response to peer review comments-- should be identified as such in the Methods section of the paper, with rationale.

**Response: We did not have a predocumented analysis plan (option b) above). We have added a mention of this to the first paragraph of the Methods and clarified when analyses were planned. We have also noted any changes in the analyses (e.g. due to reviewer comments).**

9)      Please ensure that the study is reported according to the STROBE guideline, and include the completed STROBE checklist as Supporting Information.  Please add the following statement, or similar, to the Methods: "This study is reported as per the Strengthening the Reporting of Observational Studies in Epidemiology (STROBE) guideline (S1 Checklist)."
The STROBE guideline can be found here: http://www.equator-network.org/reporting-guidelines/strobe/
a)      When completing the checklist, please use section and paragraph numbers, rather than page numbers.

**Response: We have completed the STROBE checklist and included it as Supporting Information. We also state this in the first paragraph of the Methods.**

10)     How was race/ethnicity defined and by whom?

**Response: We had no measure for race/ethnicity in our analyses because this information is not registered in Finland. Our measure of ‘Foreign background’ was based on the definition used by Statistics Finland in which persons whose both parents or the only known parent were born abroad are considered to be of foreign background. We additionally defined persons who were themselves born abroad as having a foreign background and excluded from the definition all those whose native language was Finnish or Swedish (i.e. one of the main official languages of Finland).**

11)     Please provide p values in addition to 95% CIs in the main text and tables

**Response: We have now provided p values in the main text and tables.**

12)     Throughout the text, please remove language that implies causality, such as “impact” , “predict” in the discussion and conclusion sections. Refer to associations instead.

**Response: We have revised the text accordingly.**

13)     Please provide the meaning of the bars and whisker in your figures.

**Response: We have revised the figures accordingly.**

14)     References:
a)      Please select the PLOS Medicine reference style in your citation manager. In-text reference call outs should be presented as follows noting the absence of spaces within the square brackets, e.g., "... services [1,2]."
b)      References should have no more than six names. For those with more than six names, please ensure that et al., is inserted after six names

**Response: We have revised the references accordingly.**

Comments from the reviewers:

Reviewer #1: "Income differences in COVID-19 incidence and severity: a nationwide register study of over four million individuals nested in households" analyzes Finnish national data, primarily towards identifying the effect of income differences on COVID-19 incidence and severity. The main conclusions were that low household income was a strong risk factor for both COVID-19 incidence and case severity, which is further highly attributable to a foreign background (Line 252). Particular strengths of the study include the availability of total population register data at the household level, and fairly complete data on common risk factors. The findings generally collaborate previous work on the impact of low household income on COVID-19 infection risk. Nevertheless, a number of points might be clarified:

1. In Line 96, it is stated that the main analyses are on COVID-19 infections (amongst the full population), and risk of severe illness (only amongst the infected). It might be clarified further as to whether the risk of severe illness amongst the full population would be a relevant analysis, and if so, it might be considered to be undertaken.

**Response: The reason for analyzing risk of severe illness among the infected was to focus on *case severity* i.e. the risk of severe illness once infected. Assessing the risk of severe illness in the full population, as has been done in most previous studies, conflates the risks of being infected and getting a severe illness once infected and thus makes it difficult to assess what the income differences are for case severity. However, to make our results more comparable with previous studies assessing hospitalization and mortality in the full population, we have now re-estimated the severity models for the full <65 population as a sensitivity analysis and present these results in Table S4. As expected, these results reflect the income differences in both incidence and case severity and estimates thus mostly fall between the two. These results are now briefly discussed in the last paragraph of the Results.**

2. In Line 128, it is stated that severe illness was defined as inpatient care lasting at least three consecutive days with a primary or secondary diagnosis of COVID-19. Sensitivity analysis (with accompanying demographics presented) for primary vs. secondary diagnosis might be considered, if possible.

**Response: This is an excellent point and we ran a sensitivity analysis including only primary COVID-19 diagnoses. These are presented in Table S3. The results were highly similar to our main analyses including both primary and secondary diagnoses. We briefly mention this in the second last paragraph of the Results.**

3. In Line 135, it is stated that household size was categorized as 1, 2, 3 or 4+. From Supplementary Table 1, 4+ household size accounts for 36.8% of the population at risk. Sensitivity analysis at higher granularities (i.e. 5+, 8+, etc.) might be considered if the data is available.

**Response: 4+ households indeed cover a large proportion of all households. We have now reclassified the household size to 1, 2, 3, 4 or 5+ in all our analyses. This had little impact on our main findings, but revealed an even stronger excess risk in the largest households (5+).**

4. In Line 181, it is stated that two-level logistic regression models (individuals nested in households) was used. A brief explanation of the construction/implementation of such two-level models might be provided, possibly in supplementary material.

**Response: We have now clarified the construction of the two-level models in the second paragraph of Analysis strategy in the Methods.**

5. In Line 186, it is stated that for incidence, Models 2 to 5 were built on Model 1 (Table 1). However, Table 1 appears to show missing values for entire groups for Models 2 to 4 in particular (suggesting that they may not be entirely "built on" Model 1?), and it is not immediately clear as to exactly what was included/adjusted for, in each of these models (as also relating to the previous point). As such, the full definition of the various models in Tables 1 & 2 - including any interaction variables - might be provided in supplementary material (and perhaps briefly clarified in the main text), if possible.

**Response: We have revised the text to clarify the definition of our different models in the first paragraph of Analyses of incidence in the Methods:**

**“In Model 1 we assessed the risk for COVID-19 infection by household income controlling for age (linear and squared to account for non-linear effects), sex, and regional characteristics. We also estimated a similar model — including age, sex, and regional characteristics but excluding household income — separately for each of the other COVID-19 risk-factors: household size, each household-level indicators of work and school exposure, and foreign background. These models provide crude baseline associations between each risk factor and COVID-19 incidence. We then built on the first model with household income, separately adjusting for household size in Model 2, for all household-level indicators of work and school exposures in Model 3, for foreign background in Model 4, and finally for all the variables simultaneously in Model 5.”**

**Furthermore, the tables now include footnotes describing all the covariates in the specific models.**

6. The choice of which categories/groups adjusted for in each model (as in Tables 1 & 2) might be explained further, if possible. For example, while foreign background was adjusted for and found to account for much of the effect from income differences, it appears possible that other risk factors such as urbanicity (urban with consistently significantly higher risk, may also be expected to correlate highly with foreign background) might have similar effects.

**Response: We have now added a new section in the beginning of the Methods section explaining our analytical strategy and the choice of the variables adjusted for in each model. We consider age, sex and regional characteristics as basic demographic confounders, the effects of which are not in our main focus. We thus control for them in the first model to obtain a crude baseline association between income and COVID-19 outcomes. We then control for other covariates (foreign background, household size, occupational exposures etc) one by one, in order to explicitly assess how controlling for each of them affects the income differences in COVID-19 outcomes. This gives us an idea on the social processes behind income differences in COVID-19 outcomes. As we show, the income differences in both incidence and case severity are strongly attenuated when controlling for foreign background, suggesting that it is in fact the higher proportion of people with foreign backround among low-income households that is driving the associations. We hope our analytical strategy is now more clear to the reader.**

7. In Line 317, the limitation of laboratory-confirmed COVID-19 cases likely not representing the full incidence, and potential bias in the distribution of laboratory-confirmed cases, was acknowledged. Related to this, the expected coverage of the national register used for incidence/severe illness might be briefly described. In particular, does the register cover all relevant medical facilities, or does it possibly exclude some categories, e.g. private clinics?

**Response:** **The national registers used in our study cover COVID-19 test results and patient data from both public and private sector health care providers. We have now clarified this in the third paragraph of the Methods.**

8. For Tables 1 & 2, the commonly-recognized major risk factors of age and sex appear not to have their odds ratios included. This might be addressed if possible (with accompanying demographics included in Supplementary Table 1)

**Response: We present income differences in incidence and severity by sex and age in Figures 2. and 3. However, in the relatively large Tables containing estimates for various other risk factors we decided not to show the ORs for these already well-established risk factors. The ORs for the age estimates from a model with both age and age squared are also difficult to interpret.**

9. For Tables 1 & 2, the top row might be captioned as being the Model(s), for greater clarity.

**Response: We have now captioned the top row as being the Models in all tables.**

10. From Figure 2, COVID-19 incidence is significantly lower in 65+ households, as compared to <65 households. This observation might be briefly commented on, if possible.

**Response: In response to this comment, we briefly commented the incidence differences between <65 and 65+ households in the first paragraph of Results section.**

Reviewer #2: This is an interesting and important study.
I do have the following question and comment:
Why the time range of the two cohorts is different?

**Response: The time ranges are limited to the latest available data, which was the end of 2020 for the hospital visits and 22 Feb 2021 for the COVID-19 test results. We have now clarified this in the second paragraph of the Methods.**

The length of hospital stay is not a criterion for COVID-19 severity. There are other clinical criteria the most important of which is saturation level. With regard to this I think it is better to analyze incidence of hospitalization rather than incidence of severe illness.

**Response: We agree that the length of hospital stay is not as such a criterion for COVID-19 severity. In lack of clinical information we used the length-of-stay of 3 days or more as an indicator for case severity. This was to ensure that we capture cases severe enough to warrant continuous inpatient care and exclude very short stays, possibly due to other health conditions. However, in response to this comment, we reran the severity analyses with hospitalization as the outcome (=any admission to the hospital with a COVID-19 diagnosis), and present the results in Table S2. Defining the outcome as any hospital visit increased the number of severe cases by about 10%, but had little impact on our results. We briefly comment on these and other sensitivity analyses in the last paragraphs of the Results.**

Reviewer #3: Overall comments:
This study examines a national data in Finland to investigate the association between household income and risk of COVID-19 infection and severe illness. A major strength of this manuscript is the database that they are using, which includes data on all individuals in Finland. In general, most of the manuscript is written appropriately. My main critique is that I think the analyses and writing need to be framed and much more rooted in modern causal epidemiologic terms. This involves thinking carefully about how adjustment is done and what the interpretation of each analysis. Many of the analyses seem to be focused on trying to understand mechanisms so formal mediation analyses may actually be the most appropriate. The strategy forward for doing this I think is conceptualizing the hypothesizing causal mechanisms using perhaps DAGs. This will help sharpen the exact questions being answered, how the analyses should be designed, and the appropriate interpretation of the results.

**Response: In response to reviewer 3#’s critique, we had a thorough reassessment regarding the conceptualization of our research question and analyses. We drew DAGs to illustrate the hypothesized causal relationships regarding our key question of interest, i.e. the association between household income and COVID-19 outcomes. As shown in these DAGs below (Figure A for incidence and Figure B for case severity), we do not measure mediators for this association in our analyses, as all our covariates conceptually or temporally precede household income. Thus a formal mediation analysis would not be suitable for our research question.**

**Because our analyses (and correspondingly our DAGs) lack any causal mechanisms from income to COVID-19 outcomes, we think they are not very well suited for illustrating our research questions. We are thus somewhat doubtful on the added value of presenting the DAGs for clarifying our analysis and suggest leaving them out of the manuscript. However, we have made changes throughout the manuscript in order to clarify our analytical strategy, and included a separate section ‘Analytical strategy’ in the beginning of the Methods section. We have revised the language throughout the manuscript in order to clarify the role of the sociodemographic covariates as confounding factors in the association between household income and COVID-19 outcomes.**

**Figure A. Directed acyclic graph illustrating the associations in the analysis of COVID-19 incidence. Key association of interest in red.**

**Figure B. Directed acyclic graph illustrating the associations in the analysis of COVID-19 case severity. Key association of interest in red.**

Also, in general, I think the association with being foreign born came out very strongly but this relationship was not thoroughly explored.

**Response: Having a foreign background indeed came out as a strong risk factor for both incidence and case severity. Furthermore, in response to the suggestion of reviewer #3 we included an analysis on the interaction between household income and foreign background. This analysis further highlighted the importance of foreign background in bringing about the income differences in COVID-19 outcomes. We have addressed these findings in the third and fourth paragraphs of the Discussion section under the heading Comparison with other studies. However, as our main research question is the association between income and COVID-19, we think that a more thorough analysis of the association between foreign background and COVID-19 remains an important topic for future studies. For these studies a formal mediation analysis would be an appropriate choice.**

Specific comments:

Intro
*       The manuscript starts with the fact many studies have identified associations with low income and education and COVID-19, but they weren't adjusted for other covariates. I think an important question to ask is: Should they be adjusted?  There are significant association between occupation, ethnicity, household size, and income. Other covariates are not necessarily confounders but may in fact be important mediators of effect. A simple example.  Being foreign born leads to only able to acquire low income jobs leading to the need to live in multi-generational households, which leads to increased risk of COVID-19 due crowding. This manuscript (and others) by Camara Jones provides a lot insights in how to conceptualize design choices when considering disparities (<https://academic.oup.com/aje/article/154/4/299/61900>). It mostly applies to racism in the United States by a lot of the principles are applicable. In the end, these covariates are not independent biologic-like factors,
but representative of a complex societal network and system that we all live in.

**Response: We very much agree that the covariates we treat as confounding factors in our analysis may be in complex interplay with income and with each other in bringing about the COVID-19 outcomes. However, we do not believe any of the covariates we measure could act as mediators in the association between income and COVID-19, because they conceptually and/or temporally precede income (see DAGs above). We are thus not overadjusting for potential mechanisms but including antecedents of income to our analyses.[1] We control for these antecedents or confounders in order to understand why the income-COVID-19 association comes about: whether there are other important risk factors for COVID-19 that are concentrated in low-income households and that can explain the associations. We have revised the Introduction and the Discussion to be more clear on our aims and rationale. We have also added a new section Analytical Strategy in the Methods to explicate the role of the covariates in our analyses.**

*       The introduction is written okay, but I feel like it bounces around a lot. I get the sence that the questions of interest relate to household income and context in understanding COVID-19 transmission, but I do not come away understanding the specific questions or actual hypotheses. I think the focus of the first two paragraphs needs to be sharpened.  The third paragraph is clear.

**Response: In response to this comment, we have revised the whole Introduction in order to clarify our research questions and our rationale.**

Methods:
*       A major major strength of this paper is the data that is being used. It contain information on all individuals and households in Finland.

**Response: We agree that this is a major strength and contribution of the study.**

*       I somewhat understand the rationale for only including households with working-age individuals, but in general the older population is the most concern for COVID-19. There still is something to be said about COVID risk in older households too. They are not likely isolated and inclusion could perhaps yield some insights about COVID acquisition through occupational hazards vs. non-occupational.

**Response: We agree with the general observation that the older population is the biggest concern with COVID-19, particularly in terms of severity. However, our main research question addressed income differences in COVID-19 outcomes, and whether different individual-level and household-level risk factors explained these differences. We restricted the analyses to under-65 population because the distributions of the risk factors on the one hand (e.g. most over-65 households are in the lower end of the income distribution, with no work or school exposures to social contacts etc.) and outcomes on the other hand (e.g. over-65 households with few infections but large risk for severe COVID-19) were so different across the age groups, that the results from analysis combining these two age groups would have been very difficult to interpret. Furthermore, because 94% of COVID-19 infections during the study period were reported in under-65 households, analyzing the older population separately was not statistically feasible. We now justify our focus on the <65 population in the first paragraph of Analytical Strategy in the Methods section.**

*       I think more clarity on the rationale for selecting covariates and designing analyses are needed. In general, I would strongly advocate for drawing a DAG (directed acyclic graph) to formalize the hypothesized causal mechanisms at play. This also helps formalize how analyze should be design and how to handle adjustments.

**Response: We have revised the Introduction and Discussion in order to clarify our research questions and our rationale. We have also added a new section Analytical Strategy in the Methods to explicate the role of the covariates in our analyses. Please find above the DAGs reflecting our research questions.**

*       For example, when invoking mechanism I would think more about thinking about mediation analyses and trying to understand direct and indirect effects. This approach handles covariates differently then if one thinks they are confounders. I think the authors in general should consider carefully whether some of the analyses should be changed to formal mediation analyses (either causual mediation or standard mediation approaches would be appropriate).

**Response: In our view, none of the covariates we measure could act as mediators in the association between income and COVID-19, because they conceptually and/or temporally precede income. Thus a formal mediation analysis would not be suitable for our research question.**

*       I am not sure I understand the rationale of the different models that add different covariates. Again, I would try to incorporate some more causal framing to designing the analyses. What question gets answered in each model?  What questions get answered by comparing Model 2 to 3, for example.  Is an unadjusted vs. adjusted analysis all that is needed?  Again the question of whether covariates are confounders or mediators in a particular analysis is also not answered, and I think a major question here (or at least for interpretation).

**Response: We have added a new section Analytical Strategy in the Methods to explicate the role of the covariates in our analyses and the rationale behind the different models.**

*       I would avoid the using "predict".  These aren't really prediction models (which would be cross-validated to assess prediction performance) but rather identifying associations. I think also the word "effect" is used inappropriately throughout the results when most things are associations.

**Response: This is a good point. We have removed these terms from our manuscript and talk about associations instead.**

*       Use of multi-level models is appropriate. It very much like the use of intraclass correlations for households.

**Response: Thank you for this comment. We agree that the intraclass correlations for both incidence and case severity are an important addition to the current literature. We highlight these in the Abstract as well as in the Author Summary.**

Results
*       I see in paragraph 2 of the results how the different models were interpreted. It is not very clear what it means to say that being foreign born "explained" the higher incidence.  Again, I think the language needs to be framed in modern causal epidemiological terms and concepts like mediation. For example, the fact that association with income was attenuated with foreign background I think might suggest that income is a mediator of the impact of being foreign born (because the causal relationship can't go the other way) that one is mediating. If only interesting at looking at the impact of income, it is appropriate to adjust foreign born as a confounder too though.  But this clarity on what the results mean does not come through.

**Response:** **We have revised the text throughout and refer to all our covariates as confounders in order to be more clear. We have also added a new section Analytical Strategy in the Methods to explicate the role of the covariates in our analyses and the rationale behind the different models already for this to be clear to the reader already before the Results section.**

*       Rather than an interaction between income and household size…I would be most interested in income and foreign born interaction. I think this should be seriously considered.

*       The thing that stands out the most to me in Figure 4 is how the relationships change once adjusting for foreign background. This to me suggests important aspects of the relationship between foreign background, income, and household size. Again assessing the interaction with being foreign born seems like it would be quite interesting.

**Response: We thank the reviewer for these excellent suggestions. In response to these two comments we have included new analyses with an interaction between household income and foreign background for both incidence and case severity. These analyses show that the income gradient in COVID-19 incidence in the general population, in fact, arises fully from the group with foreign background. Among those with Finnish background the income gradient in COVID-incidence was non-existent. Due to the relatively small number of severe cases even in our large population-based data the interaction analyses were underpowered for case severity, and thus little can be said about them. We discuss these findings in the first and fourth paragraphs of the Discussion.**

*       It would be interesting to understand the impact of neighborhood socioeconomic impacts as will.  There are some basic descriptors of neighborhood (e.g., urban). Neighborhoods are often segregative into areas with great heterogeneity in terms of social vulnerability, but I don't these variables capture that.

**Response: The impact of neighborhood-level socioeconomic factors is indeed an interesting topic. However, we decided to focus on individual and household-level risk factors because area-level characteristics are already largely investigated in previous studies. Investigating the cross-level interactions between neighbourhood-level, household-level and individual-level risk factors would be an fruitful avenue for future research but is beyond the scope of the current study.**

*       I would more clearly label the lowest and highest income quintiles throughout.

**Response: We have now labeled the quintiles as suggested.**

*       What about clustering that happens at the neighborhood level and important neighborhood level covariates?

**Response:** **The impact of neighbourhood-level socioeconomic factors as well as neighbourhood clustering of COVID-19 outcomes is an important topic but we feel that it is beyond the scope of the current study. Particularly as the strength of the current data lies in the individual and household-level measurement of risk-factors unavailable for most previous studies relying on neighbourhood-level data.**

Discussion
*       I think in general more contextualization is needed in terms of what the findings mean beyond the different analytic strategies (e.g., this analysis was adjusted this one was not). What is larger of importance of what the results mean because of these analytic differences.

*       For example, I think more contextualization is needed in terms of this finding "In fact, the higher risk of severe COVID-19 in low-income households seems to be strongly driven by personal occupational status and a foreign background." Why would this be the case? What are the mechanisms

**Response: In response to the two comments above, we have revised the Discussion to contextualize and explicate the added value of our adjustments:**

**“Personal occupational status is likely to capture health-related confounding by controlling for being on early-age pension or having an unknown personal occupational status. Foreign background, in turn, may capture ethnic differences in the risk for severe COVID-19, the reasons for which are likely to be multifaceted and currently not very well understood.[31] One reason may relate to selection bias caused by differential testing.[32] If people with a foreign background tend to test less for mild COVID-19 symptoms, this will lead to a disproportional share of severe cases among those identified as infected in this group. Further studies should investigate the mechanisms producing social and ethnic differences in case severity, preferably in samples where infections are identified by screening rather than by self-selective testing.” (Fourth paragraph under the heading ‘Comparison with other studies‘)**

**References**

1.	Schisterman EF, Cole SR, Platt RW. Overadjustment Bias and Unnecessary Adjustment in Epidemiologic Studies. Epidemiology. 2009;20: 488–495. doi:10.1097/EDE.0b013e3181a819a1
