# Supplementary material for: Income differences in COVID-19 incidence and severity in Finland among people with foreign and native background: A population-based cohort study of individuals nested within households
Source: PLoS Med. 2022 Aug 10;19(8):e1004038. doi: 10.1371/journal.pmed.1004038 (PMC9365184; doi:10.1371/journal.pmed.1004038)
Supplement: Attachment — Submitted filename: Response_to_reviewers.docx [file pmed.1004038.s008.docx]

**Response to editors and reviewers**

We thank the reviewers for the comments on our manuscript and further suggestions for improvement. Please find below our point-by-point response to each of the issues raised by the reviewers.

Reviewer #1: We thank the authors for addressing our previous concerns, as well as the point raised by Reviewer #3 on adjustment for other covariates. The manuscript appears clearer on the whole. However, in Table 2, The second "Model 5" might be "Model 6".

**Response: We thank reviewer#1 for the comment and agree that our manuscript is now clearer. We corrected the label in Table 2.**

Reviewer #3: Overall comments:

My main critique is that there still seems to be a bit of a disconnect between what the authors think is important to analyze with regards to socioeconomic drivers of COVID-19. In the intro and discussion, they talk about broader socioeconomic drivers, many of which could be the root causes of the disparities seen in COVID-19 outcomes (and also the reason there is an association with income and COVID-19). They also seem to discuss in essence income as a mediator of being foreign-born.  However, the analysis focuses on the independent association of income with COVID-19, yet there isn't as much discussion about the important mechanisms of income that are independent of the other confounders. Thus, the focus on income when the results seem to point to the fact that there actually other more important drivers at play (being foreign-born in particular…and income as a mediator of that) is a bit confusing and should be sharpened and clarified. The reason to focus on income (and only income) in this setting needs to clear and compelling (currently I generally find the confounders more compelling and there is also more discussion of them as well).

**Response: We thank the reviewer for the thoughtful comments. We have now revised the text in order to clarify our aims and focus in the Introduction and the interpretation of our results in the Discussion. The starting point and basis of our study was the result of earlier studies suggesting a socioeconomic gradient in COVID-19 outcomes, i.e. the finding that both the infections and severe illness are more common among people with lower socioeconomic positions (as measured by income, education, and area deprivation). Because of the limitations of previous studies, it was unclear before our study, if income - one important indicator of socioeconomic position - drives COVID-19 outcomes independently of other established risk factors.**

**We considered it plausible that the socioeconomic gradients found in previous studies could arise from factors such as household size, foreign background, and intra-household transmission due to differing exposures for infection outside the home, but on the basis of previous research we could not predict the relative importance of these factors. Therefore, a better understanding of the role of these other risk factors as potential drivers of income differences was (along with the independent effect of income) the central focus of our study. We concentrate in the Introduction on these household-level exposures because we consider the lack of a household-level approach a key limitation of previous studies. We have now reformulated the Introduction in order to make this focus more clear.**

**We agree that the discussion on the independent association of income was eventually obscured by the discussion on other risk factors that we found to be important drivers of the income gradient in COVID-19 outcomes. We have therefore now revised the text and added a discussion on the potential explanations for the higher COVID-19 incidence among people with lower income and foreign background - the only independent association of income with COVID-19 outcomes that we found.**

Specific comments:

Intro

*       "thus it remains unclear whether the worse outcomes reflect socioeconomic differences in incidence, case severity, or both" - I think this could be more specific by saying whether socioeconomic status influences risk of exposure and infection, outcomes once some is infected, or both.

**Response: Thank you for this suggestion, we revised this line to: “However, as many studies assess these outcomes among the general population as opposed to the infected[1-3], it remains unclear whether socioeconomic position influences the risk of exposure and infection, or case severity, i.e. outcome once infected, or both.”**

*       Also, I think socioeconomic status often encompasses a broader array of factors including income, education, occupation, social class (which is also tied with race/ethnicity) (per APA definition). If this analysis is focused on income, I would use that specifically (which is also different than wealth).

**Response: We agree that socioeconomic position is a multifaceted concept, encompassing a broader array of factors than just income. We have now clarified our rationale for focusing on household income as an indicator of socioeconomic position at the end of the Introduction and in the strengths and weaknesses section in the Discussion. However, we believe that the existing literature using other measures of socioeconomic position than income is still very relevant for our study, which is why we approach the subject from the broader view of socioeconomic differences.**

*       When reading the introduction (particularly second paragraph), I still get the sense that this analysis is focused on the broader milieu of socioeconomic factors as opposed to just income. The discussion of household clustering, occupation exposure seems to suggest that understanding their role is also important to this analysis (and then I would also add foreign-born status to that list).  However, if the goal is only assess how income-level may drive infections and severity, I think the discussion should be focused there. Household size, occupation, race/ethnicity are then just confounders.  How does income drive COVID things independent of household size, occupation, race/ethnicity?  Is it related to underlying health access (which could lead to more comorbidities and then also worse COVID outcomes)?  Access to information?  In general (and I imagine particularly in Finland where there is a strong public healthcare system), the financial component is less the issue, but income may be an important proxy for several other risk factors (as the authors note).

**Response: This comment relates closely to the overall comment as well as the previous comment, so please also see our responses there. We have now revised the text in order to clarify our aims and focus in the Introduction and the interpretation of our results in the Discussion. In the Introduction we now more clearly state that, according to the available research, it was unclear if income (as one important indicator of socioeconomic position) affects COVID-19 outcomes independently of household size, occupation or race/ethnicity, and thus investigating this was the aim of the current study. In the Discussion we have added a section assessing how the combination of low income and foreign background may drive COVID-19 incidence independent of household size and work and school exposures.**

Methods:
*       The authors state they want to focus in on household level factors, but neighborhood-level factors are also a likely important confounder to consider when considering the relationship between income and COVID outcomes. If available, I would consider how it could be incorporated (assessments of clustering at the neighborhood level are also still interesting). If unavailable, that is fine, but I wasn't convinced by the rationale for not including them. Ultimately, they likely represent important confounders, and if unavailable, should be included as a limitation.

**Response: We explored the possibility of including neighbourhoods as a third level in our multilevel analysis. However, due to the relatively small number of infections and severe cases even in our total population data, the models would not converge. Assessing the neighbourhood clustering and risk-factors was thus not statistically possible and remains a question for better-powered future studies. We have now addressed the lack of controls for neighbourhood-level confounding as a limitation in the Discussion.**

*       I don't think necessary to say "in response to peer review comments".  I think can just say "We conducted three sensitivity analyses."

**Response: With this wording, we are following journal guidelines: “--- changes in the analysis-- including those made in response to peer review comments-- should be identified as such in the Methods section of the paper, with rationale.”**

*       For under vs. over 65 year old households, the definitions are not clear. Were only households with everyone under 65 included?  Where were households where the main earner was less than 65, but some over 65 years olds also lived (e.g., mixed households with grandparents)?  I understand excluding households where everyone is over 65 years old, but this latter group seems like they should still be included (and perhaps they are).  I think the definitions need to be clarified and also needs clarity of where these mixed households fit in. This composition is even an interesting covariate.

**Response: Under-65 households comprise all members of households with at least one person under the age of 65 at the end of 2019. Mixed households are thus included in our main analyses. The definition for under-65 households is in the second paragraph of Methods, under heading Setting and study population. We have now further clarified this in the section on Analytical strategy and the beginning of the Results section. Multi-generational households are relatively rare in Finland, and the proportion of under-65 households with a household member over the age of 65 was only 7.6%. We did not include the presence of over 65-year-olds as a covariate in our analyses, as individual-level age was already included and we did not consider the presence of an older household member to be a risk factor for COVID-19 incidence, nor case severity.**

Results
*       Incidence models - I would create a separate sentence highlighting that when foreign background was included as a covariate, the association of income and COVID-19 was largely attenuated.  As presented, seems like a secondary point.

**Response: Good point. We created a separate sentence as suggested.** **It is now: “However, after including foreign background the association of income and COVID-19 was largely attenuated (Model 4), with only the estimate of the lowest quintile (OR 1.23, 95% CI 1.16–1.30, p<0.001) remaining statistically significant.”**

*       Is it possible to present a threeway interaction between income, foreign-born, and household size.  When I see the interaction between household size and income, the first question that I have…are foreign born individuals more likely to have larger household sizes. And then the household size interaction ends up still being related to foreign born status too. This may or may not be true but I think a question that should be answered in someway.  If space is an issue, could consider only included the adjusted model in the figures and also figure 6 and 7 could potentially be supplementary.

**Response: We tested for a three-way interaction between income, foreign background and household size in the full model and found no statistically significant interactions for incidence (p=0.29) or case severity (p=0.98). This may, again, be related to lack of power to detect interactions, so firm conclusions cannot be made. We thus feel that presenting these results would add little to the current evidence, and suggest not showing these results.**

*       Even without a mediation analysis, it is clear that income is an important mediator of being foreign born it seems (agree that there aren't measured mediators of the effect of income on COVID). But it does not mediate the full effect as even among the highest income, there are differences between native Finnish and foreign-born. This piece gets lost for me.

**Response: We agree with this notion. However, this study is about the association of income with COVID-19 outcomes, with an additional analysis on how this association differs between people with native or foreign backgrounds. To keep our focus clear, we wish to avoid a larger discussion of the main effects of foreign background.** **We have also revised the Discussion to focus more clearly on foreign background in the context of the income-COVID-19 association. We believe a more thorough analysis of the association between foreign background and COVID-19 merits a separate paper and remains an important topic for future studies.**

Discussion
*       I think leading with this sentence "Individuals living in low-income households appeared to have higher  odds of both COVID-19 incidence and severity" ends up being somewhat misleading (even though it is clarified later). I think needs to also include that is specific for foreign born individuals only and not among native Finnish. The point is there is an income gradient among foreign born and there is not among native Finnish.

**Response: We agree with this point. However, we feel it is important to show that, on the population level, we indeed find the same income gradient as in the previous literature, but that when delving deeper into this general association, we find that it is strongly driven by other risk factors and, in fact, only existent in the population with foreign background. We have now reformulated the sentences as follows:**

**“In line with prior evidence, our descriptive analyses showed that, in the total population, individuals living in low-income households had higher risk of both COVID-19 incidence and severity. However, the 70% higher incidence among lowest-income households was largely driven by the foreign background of household members. In fact, a separate analysis revealed a strong income gradient among individuals with foreign background only, and no income association at all among those with native background.”**

*       I also don't know that I would say that this relationship is largely confounded by foreign born. Though technically true, that implies there was a false association (which I don't think is the authors' point). I think I would try to clarify what the results indicate, which to me is that being foreign-born is a major driver of differences in outcomes, and the income mediates some but not all of it (and also that there is no income gradient among native Finnish).

**Response: We agree with this point as well, and we have revised the sentence as follows: "However, the 70% higher incidence among lowest-income households was largely driven by the foreign background of household members. In fact, a separate analysis revealed a strong income gradient among individuals with foreign background only, and no income association at all among those with native background.”**

*       The response to reviewers and analysis seems to emphasize that the point of this analysis is to assess the association of income with COVID-19 outcomes that is independent of other socioeconomic factors such as household size, occupation, foreign-born, etc.  However, the intro nor the discussion really dives into the important independent mechanisms of income.  Most of the discussion actually seems to discuss income as a mediator for these other more proximal variables. This is the basis for my previous comments that it might be prudent to also have some focus on income as mediator for these more proximal variables (as opposed to mediators of income itself). But if the focus is on the independent effects of income, there needs to be discussion of those specifically.

*       To be fair, my thinking generally is in line with the authors framing of the broader socioeconomic milieu being very important drivers, with income as one of the mediators of them.  But the stated focus of the analysis is somewhat contrary to that, so that discussion needs to be there.

*       For example, "Taken together, our results suggest that income is an independent risk factor for COVID-19 incidence only among people with foreign background. The disadvantage among those with foreign background and low household income may relate to the work and school exposures, household size, and comorbidities that we were able to measure." These sentences combine income as mediator of foreign background (along with household composistion, work, school) and the independent mechanisms of income alone.  The discussion of how income may independently relate to COVID-19 outcomes is lacking.

**Response to the three comments above: These comments relate closely to the overall comment as well as some of the previous specific comments, so please also see our responses there. We have now revised the text in order to clarify our aims and focus in the Introduction and the interpretation of our results in the Discussion. In the Introduction we now more clearly state that, according to the available research, it was unclear if income (as one important indicator of socioeconomic position) affects COVID-19 outcomes independently of household size, occupation or ethnicity/foreign background, and thus investigating this was the aim of the current study. As we found an independent association between income and COVID-19 incidence only among people with foreign background, we have therefore focused the Discussion on the mechanisms bringing about the income gradient in this group specifically:**

**“Our results suggest that, overall, there is no independent association between household income and COVID-19 incidence. Low income was related to higher COVID-19 incidence only among people with foreign background, and this association was independent of household size and the work and school exposures that we measured. The combination of foreign background and low income is likely to capture vulnerabilities related to race, ethnic minority, and refugee background which may influence infection risk through material, social, and behavioural mechanisms. Prior studies from the US indicate that people with low income have fewer material and social resources to protect themselves from COVID-19 infection.[27] Social mechanisms may relate to lower health literacy, language barriers, racism, and structural discrimination faced by people with foreign background and low socioeconomic position.[28–30] Higher susceptibility to COVID-19 may also relate to a lowered immune response due to higher stress-levels.[14,31] Finally, behavioural factors such as broader social interactions among people with foreign background [29] may further add to the clustering of risk factors among those with foreign background and low income. Due to the epidemic nature of COVID-19, the extent of social interactions could be of particular importance if outbreaks are concentrated in schools and neighborhoods having more people with low socioeconomic position and foreign background. The lack of neighborhood-level controls is a limitation of our study.”**

*       "Foreign background, in turn, may capture ethnic differences in the risk for severe COVID-19, the reasons for which are likely to be multifaceted and currently not very well understood".  I am not sure I agree with this sentence. First, it does seem to imply a biologic reason for differences (which I am not sure the authors intended). Second, I do think we know a lot about why being foreign-born person relates to COVID-19, many of which the authors do discuss throughout.  But it relates to structural racism, issues with access (which could be driven by language, education, access to information), socioeconomic factors, neighborhood deprivation, etc. There has been a lot of literature about drivers of health disparities (even prior to COVID), and many probably are also relevant to Finland. This discussion could be more robust and perhaps combined with the discussion in the preceding paragraph.

**Response: We agree that the literature has identified a plethora of possible reasons for the ethnic differences in health. However, according to a recent study in the Lancet, the evidence on specific mechanisms for COVID-19 seems still to be rather fragmented and suggestive.[1] We have now revised our wording, but in order to keep the Discussion more tightly focused on income, we have aimed to be brief in referring to this literature. Furthermore, as both the results as well as the mechanisms differ for COVID-19 incidence and case severity, we prefer to keep the discussion of these separate. See our response to the comment above and changes to the 4^th^ and 5^th^ paragraph of the discussion.**

**References**

1.	Mathur R, Rentsch CT, Morton CE, Hulme WJ, Schultze A, MacKenna B, et al. Ethnic differences in SARS-CoV-2 infection and COVID-19-related hospitalisation, intensive care unit admission, and death in 17 million adults in England: an observational cohort study using the OpenSAFELY platform. The Lancet. 2021;397: 1711–1724. doi:10.1016/S0140-6736(21)00634-6
