# Supplementary material for: Income differences in COVID-19 incidence and severity in Finland among people with foreign and native background: A population-based cohort study of individuals nested within households
Source: PLoS Med. 2022 Aug 10;19(8):e1004038. doi: 10.1371/journal.pmed.1004038 (PMC9365184; doi:10.1371/journal.pmed.1004038)
Supplement: Attachment — Submitted filename: Response_to_reviewers.docx [file pmed.1004038.s009.docx]

**Response to editors and reviewer**

We are pleased to hear that our manuscript has improved and is planned to be accepted for publication. We thank the editor and the reviewer for the comments and further suggestions. Please find below our point-by-point response to each of the issues raised by the editor and the reviewer.

Any changes to the reference list were asked to mention in the rebuttal letter. We have added the following references to the manuscript since the initial submission:

[13] Vandentorren S, Smaïli S, Chatignoux E, Maurel M, Alleaume C, Neufcourt L, et al. The effect of social deprivation on the dynamic of SARS-CoV-2 infection in France: a population-based analysis. Lancet Public Health. 2022; S246826672200007X. doi:10.1016/S2468-2667(22)00007-X **(lines 101, 116, 478)**

[14] Khanijahani A, Iezadi S, Gholipour K, Azami-Aghdash S, Naghibi D. A systematic review of racial/ethnic and socioeconomic disparities in COVID-19. Int J Equity Health. 2021;20: 248. doi:10.1186/s12939-021-01582-4 **(lines 101, 478)**

[27] Clouston SAP, Natale G, Link BG. Socioeconomic inequalities in the spread of coronavirus-19 in the United States: A examination of the emergence of social inequalities. Soc Sci Med. 2021;268: 113554. doi:10.1016/j.socscimed.2020.113554 **(line 426)**

[31] Myers HF. Ethnicity- and socio-economic status-related stresses in context: an integrative review and conceptual model. J Behav Med. 2009;32: 9–19. doi:10.1007/s10865-008-9181-4 **(line 430)**

[32] Williams DR, Lawrence JA, Davis BA, Vu C. Understanding how discrimination can affect health. Health Serv Res. 2019;54: 1374–1388. doi:10.1111/1475-6773.13222 **(line 452)**

[33] Pan D, Martin CA, Nazareth J, Nevill CR, Minhas JS, Divall P, et al. Ethnic disparities in COVID-19: increased risk of infection or severe disease? The Lancet. 2021;398: 389–390. doi:10.1016/S0140-6736(21)01428-8 **(line 453)**

[34] Lassale C, Gaye B, Hamer M, Gale CR, Batty GD. Ethnic disparities in hospitalisation for COVID-19 in England: The role of socioeconomic factors, mental health, and inflammatory and pro-inflammatory factors in a community-based cohort study. Brain Behav Immun. 2020;88: 44–49. doi:10.1016/j.bbi.2020.05.074 **(line 479)**

[37] Griffith GJ, Morris TT, Tudball MJ, Herbert A, Mancano G, Pike L, et al. Collider bias undermines our understanding of COVID-19 disease risk and severity. Nat Commun. 2020;11: 5749. doi:10.1038/s41467-020-19478-2 **(line 493)**

Requests from Editors:

1)      Please revise your title to include “foreign background,” considering the association between living in low-income households and COVID-19 incidence and severity is specific for foreign born individuals only and not among native Finnish.
2)      Please revise your subtitle to “…nested within households” as opposed to “…nested in households.”

**Response: We revised the title as: “Income differences in COVID-19 incidence and severity in Finland among people with foreign and native background: a population-based cohort study of individuals nested within households”.**

3)      Abstract and Results- Please include the actual amounts or percentages of relevant outcomes in addition to the ORs

**Response: We added percentages of relevant outcomes to the Abstract and Results.**

4)      Discussion, Line 457 - Please avoid assertions of primacy (“Our study is the first to..."). Instead, state “To our knowledge, …” or similar

**Response: We revised the line as** “**To our knowledge, our study is the first to assess the household clustering of case severity.”**

5)      Please include definitions for all abbreviations in the tables, e.g., ICC

**Response: We included definitions for all abbreviations to the first line of notes below regression results in relevant tables.**


Comments from Reviewers:

Reviewer #3: Overall comments:
I thank the authors' for their efforts in addressing my comments, and I think they have provided additional clarity on several issues that were raised. There are still a few points that I think would strengthen the paper (which I list below), though I think there is likely a conceptual disagreement on how I and the authors' think they should be approached (which is okay). I only raise them again as I think causal and conceptual clarity is particularly important in discussions of disparities—in large part because of the history how of they have been inadequately discussed—but would defer to the editors and authors' on how best to handle them at this stage.

**Response: We thank the reviewer for the mindful comments and further suggestions for improvement. We have now further revised the Intro in order to improve conceptual clarity.**

Specific comments:

*       I think there is too much interchangeability between the use of socioeconomic factors and income in the introduction. As mentioned previously, I think at least some discussion that clearly differentiates the mechanisms between the two is important somewhere. They do so in discussion of the methods, though some readers may interpret a false equivalence between the two after reading the intro. I do not think the authors' intend this, but do think it is easy to miss the distinction, which is key to this paper, because they have too often been conflated for each other in the past.

**Response: We revised the Introduction to** **emphasize the income’s role as an indicator of more multifaceted concept:**

**“We use household income as an indicator for the multifaceted concept of socioeconomic position. Household income is a reliable measure available for all individuals irrespective of their age, employment or immigrant status**.**”**

*       I think the threeway interaction between income, foreign-born, and household size could still be interesting and important.  It is difficult to assess just from the p-values since it is not unexpected that p-values would be high in a threeway interaction (p=0.29 for a threeway is not too high).  When point estimates and CIs are graphed, are there any insights from the trends…is everything still driven by being foreign born? That would be the data that would inform my assessment of whether it is relevant or not. It would be great if authors' could share these results at least and could consider as supplemental figure. Again, the authors' may already done this type of full assessment, but from the reponse it seems solely based on the p-value.

**Response: We thank the reviewer for raising this issue again. We drew a graph of the three-way interaction between income, household size, and foreign background, and included it as Supplementary Figure 1. The graph indeed indicates that the association of poor and large households with COVID-19 incidence arises from population with foreign background. This is a valuable addition to our manuscript. We have made the following changes to the manuscript:**

**“In response to reviewer feedback, we further tested for the interaction between household income and foreign background, and a three-way interaction between household income, household size and foreign background.” (Third paragraph under heading ‘Analytical strategy’ in the Methods)**

**“An additional interaction analysis between household income, household size and foreign background (S1 Fig) indicated that the excess risk of large poor household was present only among those with foreign background.” (Third paragraph under heading ‘Incidence Models’ in the Results)**

**“Our results further suggest that this accumulation mainly occurs among the population with foreign background.” (Fifth paragraph under heading ‘Comparison with other studies’ in the Discussion)**

*
*       It took me a few reads to understand the point being made in the discussion by "in the total population, individuals living in low-income households had higher risk of both COVID-19 incidence and severity…" My first two reads interpreted this has trying to make a claim about some level of generalizability of the findings (and not in just an artefactual mathematical sense) even though the following statements did clarify the issue. Though I think the authors' are trying to say that they found a similar result to previous studies, but they also find that it is in fact not generazable. Could consider saying something like "Prior evidence has shown associations with income in the total population, but our deeper dive indicated that these trends were in fact driven primarily by a specific population and not generalizable to the whole population" or something of that nature.

**Response: We revised the sentence as:** **“In line with prior evidence, we found that individuals living in low-income households had higher risk of both COVID-19 incidence and severity – however this was largely driven by the foreign background of household members.” We hope that it now reads clearer.**
