# Supplementary material for: Income differences in COVID-19 incidence and severity in Finland among people with foreign and native background: A population-based cohort study of individuals nested within households
Source: PLoS Med. 2022 Aug 10;19(8):e1004038. doi: 10.1371/journal.pmed.1004038 (PMC9365184; doi:10.1371/journal.pmed.1004038)
Supplement: Attachment — Submitted filename: Response_to_reviewer.docx [file pmed.1004038.s010.docx]

**Response to the reviewer**

Reviewer #3: Thank you to the authors' for their revisions and their inclusion of a figure of the threeway interaction. I do think it really helps to emphasize the studies main findings. I appreciate the authors' clarification that they are using income as a proxy for the multidimensional construct for socioeconomic status. I think clarifications are needed in two places: in the intro and strengths and limitations.

**Response: We agree that including the three-way interaction indeed strengthened our analysis. We also want to thank the reviewer for the further suggestions for improvement. We have now revised the Introduction and the Strengths and weaknesses sections to clarify the advantages and limitations of household income as an indicator of socioeconomic position.**

When the authors say that household income is a "reliable" measure, do they mean reliable because it was available for everyone or reliable because it is an adequate proxy for this complicated multidimensional construct.  It reads like the latter, but I think the results themselves directly speak against it being the latter. I would change the wording of this in the intro.

**Response: We changed the wording: “We focus on household income as it is reliably measured and available for all individuals irrespective of their age, employment or immigrant status.”**

I also think one or two sentences need to be added to the strengths and limitations to be clear that this is a limitation (currently the wording seems to describe this as a strength).  Specifically, that they used income as a proxy for a complicated multidimensional construct, which is not ideal because socioeconomic status also includes considerations related to race, community, etc., but was done because that data was what was reliably available. The completeness of the income data is a strength but using at is a proxy for complex construct is certainly an important limitation.

**Response: We revised the Strengths and weaknesses section as: “Of the common indicators of socioeconomic position (education, income, deprivation), we chose to focus on household income. It is reliably measured and available for all individuals irrespective of their age, employment or immigrant status, and well-suited for household-level analyses. We also had access to data on annual household income from a time point close to the pandemic, 2018. However, a limitation of our study is that household income captures only one aspect of the multidimensional concept of socioeconomic position. While our study also included information on occupational status, future research incorporating multiple dimensions of socioeconomic position is needed for a more comprehensive picture of the social inequalities in COVID-19 outcomes.”**
